# Optimised production of technetium-94m for PET imaging by proton-irradiation of phosphomolybdic acid in cyclotron liquid target

**DOI:** 10.1016/j.apradiso.2024.111381

**Published:** 2024-08

**Authors:** Ross Harper, Derek R. Morim, Dhyey Mehta, Veronika Rosecker, Stephen J. Archibald, Richard Southworth, Philip J. Blower, Karin A. Stephenson, Karin M. Nielsen

**Affiliations:** a Nuclear Operations and Facilities, McMaster University, 1280 Main St W, Hamilton, ON, L8S 4L8, Canada; b School of Biomedical Engineering and Imaging Sciences, King's College London, St Thomas' Hospital, Westminster Bridge Road, London, SE1 7EH, United Kingdom; c Center for Labelling and Isotope Production, TRIGA Center Atominstitut, TU Wien, Stadionallee 2, 1020, Vienna, Austria; d Institute for Applied Synthetic Chemistry, TU Wien, Getreidemarkt 9, 1060, Vienna, Austria

**Keywords:** Technetium-94m, Radionuclide production, Cyclotron targetry, Phosphomolybdic acid, Liquid target, Positron emission tomography

## Abstract

Natural-abundance phosphomolybdic acid (H_3_(Mo_12_PO_40_) ‧12H_2_O, 0.181–0.552 g Mo/mL) solutions were irradiated with 12.9 MeV protons on a GE PETtrace cyclotron using an adapted standard liquid target. Technetium-94m (^94m^Tc) was produced through the ^94^Mo(p,n)^94m^Tc nuclear reaction with saturation yields of up to 53 ± 6 MBq/μA. End of bombardment activities of 161 ± 17 MBq and 157 ± 7 MBq were achieved for the 0.552 g Mo/mL solution (10 μA for 30 min) and 0.181 g Mo/mL solution (15 μA for 60 min), respectively. No visible degradation of the niobium target body and foil were seen during the irradiations of up to 15 μA for 60 min. The produced ^94m^Tc was separated from the target phosphomolybdic acid solution with >98% recovery using an aqueous biphasic extraction resin. Compared to previous reported liquid target methods for ^94m^Tc production, the better production yield, in-target solution stability during irradiation and ^94m^Tc separation recovery of phosphomolybdic acid makes it a very promising target material for routine clinical ^94m^Tc production at medical facilities with liquid targets already installed for ^18^F production.

## Highlights

• ^94m^Tc could bring the benefits of Tc-tracer research to the field of total body PET.
• Optimised production of ^94m^Tc in cyclotron liquid target.
• Proton-irradiation of phosphomolybdic acid with saturation yields up to 53 MBq/μA.
• Excellent Tc/Mo separation using aqueous biphasic extraction resin.

## 1 Introduction

Technetium-99m radiopharmaceuticals have been the mainstay of nuclear medicine for over half a century. The decay characteristics of ^99m^Tc (t_½_ = 6.01 h, E_γ_ = 140.5 keV (87%)) make it ideal for single-photon emission computed tomography (SPECT). However, the application of key molecular imaging ^99m^Tc-tracers, such as ^99m^Tc-exametazime and ^99m^Tc-sestamibi, although having thoroughly-studied targeting mechanisms, remains limited by the relatively poor resolution offered by SPECT imaging (Bigott et al., 2005; Ruth, 2009). The higher sensitivity and better resolution of positron emission tomography (PET) make it superior for quantitative pharmacokinetic evaluation of tracers *in vivo*. The most suitable technetium isotope for PET imaging is technetium-94m (t_½_ = 52.0 min, I_β+_ = 70.2%, E_β+,max_ = 2.44 MeV) (Gagnon et al., 2011). The switch to a positron-emitting isotope of the same element offers compatibility with currently-developed tracers and production methods for widely-used clinical ^99m^Tc-tracers with well-established applications. The advantage of the associated lower clinical pharmaceutical regulatory barrier facilitates the possibility for rapid approval of proof-of-concept PET studies. Robust production methods for ^94m^Tc-pertechnetate and other ^94m^Tc-complexes will enable applications in, for example, reporter gene imaging (Sharif-Paghaleh et al., 2011), mitochondrial function imaging (Safee et al., 2019) and the use of supramolecular cages in PET radiopharmaceutical design (Brader et al., 2013; Burke et al., 2018). Furthermore, ^94m^Tc would bring the benefits of decades of Tc-tracer research to the fast-developing field of total body PET providing quantitative dynamic imaging across the whole body.

The cyclotron production routes of ^94m^Tc have been extensively evaluated with protons, deuterons, ^3^He and ^4^He (Bigott et al., 2006; Denzler et al., 1995; Faβbender et al., 1994; Khandaker et al., 2007; Nickles et al., 1993; Rösch and Qaim, 1993). Of the nuclear reactions evaluated, the ^94^Mo(p,n)^94m^Tc is the most suitable one for small-sized cyclotrons, with an optimal proton energy range from 13 to 7 MeV giving the highest ^94m^Tc yield and purity (Hoehr et al., 2012; Qaim, 2000). The low natural abundance of ^94^Mo (9.2%) necessitates the use of highly enriched ^94^Mo and an efficient recycling procedure. Most previous studies have focused on production using solid molybdenum targets, which will not be applicable for many hospital cyclotrons, where solid targetry may not be available. In contrast, the potential to use liquid targets that are already installed at most medical facilities for clinical ^18^F production would enhance the accessibility of ^94m^Tc, especially since its relatively short half-life of 52 min limits distribution between sites. Although liquid targets deliver lower production yields than solid targets because of their lower nuclei density, they have the advantage of not requiring a dissolution step, thereby simplifying and expediting the post-irradiation processing – a key advantage for shorter half-life radionuclides such as ^94m^Tc.

In 2012, Hoehr et al. (2012) published a method for the cyclotron production of ^94m^Tc (110 ± 20 MBq) using a standard liquid target for the 12 MeV proton irradiation of ammonium heptamolybdate tetrahydrate ((NH_4_)_6_Mo_7_O_24_ ‧4H_2_O, AHM, water solubility 0.43 g/mL) solutions containing hydrogen peroxide. The authors reported issues with the concentrated AHM solutions (0.325–0.995 g/mL) causing high pressure build-up in the target even at low (5 μA) beam currents, suspected to be due to gas evolution derived from high concentration of ammonium salts. In addition, formation of salt crystals from the solution irradiations caused clogging of valves and difficulties emptying the target. High pressure build-up and solution precipitation is not uncommon for salt solution irradiations and have been reported for liquid target productions of other radionuclides (Do Carmo et al., 2020; Jahangiri et al., 2018; Pandey and DeGrado, 2021). Finally, Hoehr et al. (2012) observed flakes of the aluminium target body in the target solution after irradiation due to metal corrosion and suggested the use of a niobium-bodied target for future studies to avoid corrosion. The high corrosion resistance of niobium derives from a readily-formed, adherent, passive oxide film. Niobium, however, like aluminium, is less resistant in alkaline solutions such as sodium hydroxide and potassium hydroxide, which can lead to absorption of hydrogen and niobium embrittlement (Sutherlin and Graham, 2005).

To address the issues reported for alkaline molybdenum solutions and taking into account the excellent corrosion resistance of niobium in acidic environment, it was decided to explore the cyclotron production of ^94m^Tc by irradiation of phosphomolybdic acid (H_3_(Mo_12_PO_40_) ‧12H_2_O, PMA, water solubility 2.90 g/mL) solutions as an alternative to AHM. Compared to AHM, PMA is highly soluble in water, has a higher molar molybdenum content (PMA: 13.0%, AHM: 9.6%) and a slightly better thermal stability (Kendell and Brown, 2010; Wu, 1920). All these characteristics makes PMA a very promising target material. Furthermore, under basic conditions PMA decomposes into molybdate (MoO_4_^2−^) (Fujinaga et al., 1970), enabling the post-irradiation processing and purification of ^94m^Tc through known methods for pertechnetate (TcO_4_^−^) and molybdate separation by solid-phase extraction (IAEA, 2017). The initial evaluation of the production using PMA, presented here, provides the basis for a simple and fast procedure to produce sufficient quantities of ^94m^Tc for *in vivo* applications, using a standard liquid target and an efficient post-irradiation processing method, which can be readily adapted for clinical ^94m^Tc-tracer production.

## 2 Materials and methods

### 2.1 Cyclotron targetry

**Fig. 1:**
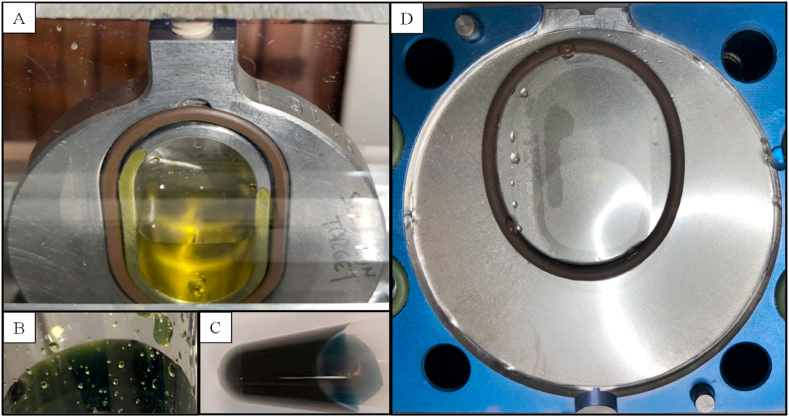
Cyclotron niobium target pre- and post-irradiation and sequential PMA colour changes during the experiments. A) Pre-irradiation, the yellow coloured (UV–vis: 420 nm) PMA solution (2.3 mL) was loaded into the niobium target closed by a transparent plastic plate to assess the volume necessary to cover beam profile area (maximum 15 mm diameter). B) The PMA solution A (0.16 mol/L) turned green when irradiated in the cyclotron up to 15 μA for 60 min. The green solution re-oxidised within 2 h back to a yellow solution. C) The PMA solution B (0.48 mol/L) turned dark blue (UV–vis: 725 nm) when irradiated in the cyclotron niobium target with 10 μA protons for 10–30 min and remained blue for months. D) Niobium foil after irradiations (n = 16) of the PMA solution A up to 15 μA for 60 min showed no visual signs of degradation or solution crystallisation. The visible liquid residue was from the post-irradiation water rinse, as the target was not dried between the water rinse steps and next production. (For interpretation of the references to colour in this figure legend, the reader is referred to the web version of this article.)

All experiments were performed on the 16.5 MeV proton PETtrace cyclotron (GE Healthcare, Sweden) at McMaster University, Canada. The irradiations were performed using a standard Nb25 liquid target (GE Healthcare, Sweden) with a water-cooled niobium body and helium-cooled entrance foils. Due to its general excellent corrosion resistance, a niobium foil (200 μm, Goodfellow, United Kingdom) was placed between the niobium body and helium, while a HAVAR foil (25 μm, GE Healthcare, Sweden) was used between helium and cyclotron vacuum, degrading the proton energy incident on the target material to 12.9 MeV. In both foils, the degraded proton energy was calculated using the SRIM program (Ziegler, 2013). The internal volume of the niobium body was approximately 3.5 mL, formed by a 10 mm deep and 3.5 cm^2^ area oval cavity. The maximum beam area on the target cavity is defined by the 15 mm inner diameter width of the target beam line. The filling volume to cover the 15 mm diameter maximum beam profile area of the target cavity was determined visually by filling the niobium body with PMA, while having the front covered using a transparent plastic plate instead of the niobium foil (Fig. 1A).

**Fig. 2:**
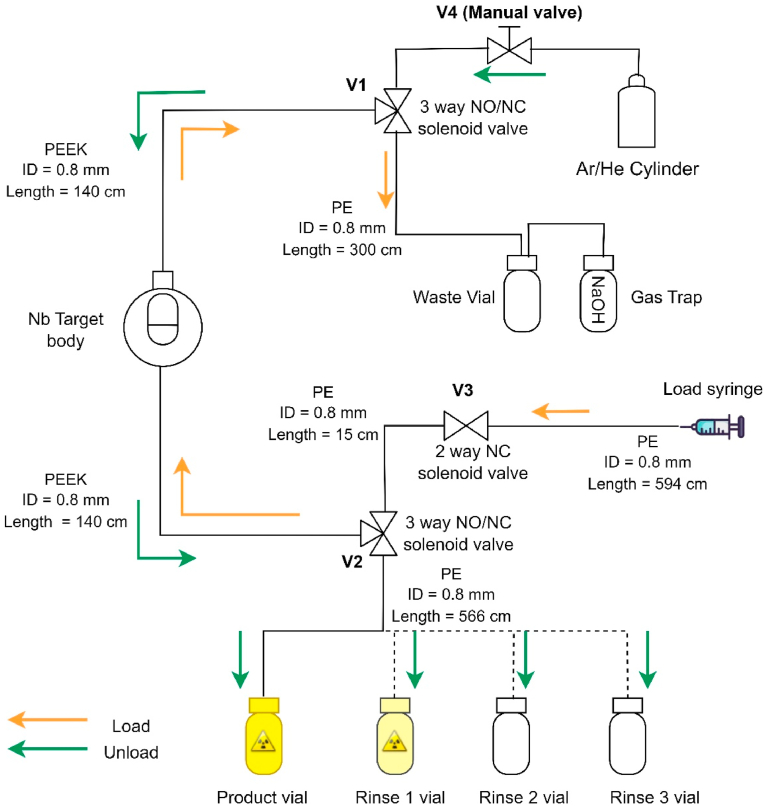
Schematic representation of targetry loading and delivery setup used for the PMA irradiation experiments. The V1, V2 and V3 2- and 3-way solenoid valves were remotely controlled through a switch box. The V4 manual shut-off valve was used for opening the either the argon or helium gas supply. Argon was used in all experiments for target emptying post-irradiation, while helium was only used for a PMA delivery test from the target to the upstairs hot cell. The target was loaded (orange arrows) from the bottom with the path through valves V1, V2 and V3 open from the manually-controlled loading syringe through the target to the waste and vented gas trap (with 1 mol/L NaOH) vials. After loading, V3 was remotely switched into its closed position for irradiation, while the target chamber was left open via the V1 valve to the waste and vented gas trap vials. The target was emptied (green arrows) by remotely switching the V1 and V2 valves into the unload position, while manually opening valve V4. The rinse steps were completed by loading water from the syringe position into the target and emptying into the rinse vials 1–3. PE: polyethylene, ID: internal diameter, NO: normal open, NC: normal close. (For interpretation of the references to colour in this figure legend, the reader is referred to the web version of this article.)

An in-house designed loading and delivery system, as illustrated in Fig. 2, was used for the experiments reported herein. The 2- and 3-way solenoid valves (V1, V2, V3, model 0127, Burkert, Germany) were remotely controlled through a switch box located in the adjacent cyclotron control room, while for the target material delivery a simple manual shut-off valve (V4, 40 Series, Swagelok, Canada) was used for opening the gas supply (argon or helium, 15–30 psi) placed in the cyclotron plant room. The lines, with dimensions as indicated in Fig. 2, were of PEEK (McMaster-Carr, Canada) between the target and the V1 and V2 valves, while the other lines were polyethylene (Natvar, Belgium). The target was loaded from the bottom with valves V1, V2 and V3 operated such that the path was open from the manually controlled loading syringe through the target to the waste and vented gas trap vials (Fig. 2). Prior to ^94m^Tc production, the operation of the target loading and delivery system was tested and optimised with both water (n = 3) and 0.48 mol/L PMA solution (n = 3). Irradiation of various volumes (1.9–2.7 mL, see Table 1) of PMA solutions was evaluated by filling the loading syringe with the PMA volume and 8 mL of air, thereby manually pushing the target solution into the target chamber. An additional 10 mL of air was pushed through the lines to remove residues of PMA before remotely switching V3 into its closed position for irradiation. During the irradiations the target chamber was left open via the V1 valve to the waste and vented gas trap vials.

**Table 1:** Cyclotron irradiation parameters for the PMA solution A (0.16 mol/L) and solution B (0.48 mol/L) and the post-irradiation ^94m^Tc activity distribution between the collected product (1.5–2.4 mL) and rinse vials (3 × 7.5 mL).

Solution	Beam current (μA)	Irradiation time (min)	Target loading volume (mL)^a^		% of total ^94m^Tc activity		
					Product	Rinse 1	Rinse 2 & 3
This work A (n = 16)	10	10	2.63 ± 0.08 2.26 ± 0.04 1.89	(n = 3) (n = 2) (n = 1)	96.7 ± 0.13 97.2 ± 2.77 94.4	3.2 ± 0.16 2.8 ± 2.77 5.6	<0.05 <0.05 <0.05
		30	2.38 ± 0.26	(n = 2)	95.9 ± 1.58	4.0 ± 1.59	<0.05
		60	2.72 ± 0.04	(n = 2)	96.9 ± 0.64	3.1 ± 0.62	<0.05
	15	10	2.61 ± 0.05	(n = 2)	95.7 ± 1.03	4.3 ± 1.03	<0.05
		30	2.65 ± 0.02	(n = 2)	95.7 ± 0.29	4.3 ± 0.30	<0.05
		60	2.64 ± 0.02	(n = 2)	95.5 ± 0.38	4.5 ± 0.41	<0.05
This work B	10	10	2.61 ± 0.02	(n = 2)	96.5 ± 0.56	3.4 ± 0.61	<0.05
(n = 4)		30	2.39 ± 0.37	(n = 2)	80.7 ± 2.26	19.3 ± 2.25	<0.05

a 81–91% of the target loading volume was recovered in the product vial for PMA solution A and 73–90% of the loading volume for PMA solution B.

The target was emptied after irradiations by remotely switching the V1 and V2 valves into the unload position, while manually opening the V4 valve to the delivery gas supply. Argon was used as delivery gas in all experiments, except for the delivery test to the hot cell described below. To rinse the target, valves and lines after product delivery, three cycles of loading 7.5 mL distilled, deionised water from the syringe position into the target and emptying into the rinse vials were performed as further detailed in Fig. 2. The irradiated target product solution and subsequent rinse steps were collected in vials placed in lead containers in the entrance corridor to the cyclotron vault. Additional transfer tests of PMA through a polyethylene delivery line (ID: 0.8 mm, approx. 20 m long) directly from V2 to a product vial placed in a hot cell on the floor above the cyclotron were also performed using helium. Detection of the delivery of the irradiated PMA into the product vial was achieved by utilising an in-house setup incorporating a semiconductor diode (Type 1N5402, Digikey, Canada) to generate a current corresponding to the intensity of the interacting gamma rays (Knoll, 2010).

### 2.2 Cyclotron target material preparation and ^94m^Tc production

Unless otherwise specified, all chemicals and consumables used were from Sigma Aldrich, Canada. Natural-abundance (^92^Mo 14.5%, ^94^Mo 9.2%, ^95^Mo 15.8%, ^96^Mo 16.7%, ^97^Mo 9.6%, ^98^Mo 24.4%, and ^100^Mo 9.8% (Wieser and Laeter, 2007)) PMA solutions (A: 0.16 mol/L and B: 0.48 mol/L) were used for the experiments reported here. For meaningful comparison, the concentrations were chosen to match the high and low molybdenum concentration solutions evaluated by Hoehr et al. (2012) (Table 2). The PMA hydrate was dissolved in distilled, deionised water and the solution adjusted to a final volume of 10 mL giving a clear yellow solution (Fig. 1A). The production of ^94m^Tc from the PMA solutions was evaluated at increasing irradiation time (10–60 min) and current (10–15 μA), as listed in Table 1, with each irradiation experiment performed in duplicate. After the completed delivery of irradiated product and rinse steps into the individual collection vials, the lead containers were transported to a lead-shielded workstation where the vials were visually inspected 20–60 min post-irradiation (Fig. 1B and C). The samples were stored in the collection vials under air in the closed lead containers until further analysis. The content of the ^94m^Tc and co-produced ^9x^Tc radionuclides in the product and rinse solutions was identified and quantified by gamma spectroscopy using an N-type HPGe detector (Ortec, United States) calibrated with a mixed ^152/154/155^Eu source. The colour changes of selected PMA samples post-irradiation were analysed by UV–vis spectroscopy on a UV-1600PC Spectrophotometer (VWR International, Canada).

**Table 2:** Composition of the molybdenum-containing PMA solutions (A: 0.16 mol/L and B: 0.48 mol/L) irradiated on the cyclotron in this work, compared to the AHM solutions A and C published by Hoehr et al. (2012).

Solution	PMA mass (g)	AHM mass (g)	Total volume (mL)^a^	Density (g/mL)^b^	Mo conc. (g/mL)
This work A	2.862 ± 0.001	–	10 ± 0.1	1.16 ± 0.01	0.181 ± 0.002
This work B	8.747 ± 0.001	–	10 ± 0.1	1.51 ± 0.01	0.552 ± 0.006
Hoehr et al. A	–	6.50 ± 0.05	20 ± 0.1	1.20 ± 0.01	0.177 ± 0.002
Hoehr et al. C	–	19.90 ± 0.05	20 ± 0.1	1.68 ± 0.01	0.541 ± 0.002

a PMA (H_3_(Mo_12_PO_40_) ‧12H_2_O) was dissolved in H_2_O (2 mL) and volume adjusted with H_2_O. For Hoehr et al. solution A & C the AHM ((NH_4_)_6_Mo_7_O_24_ ‧4H_2_O) was dissolved in H_2_O_2_ (1.0–1.2 mL) and final volume adjusted with H_2_O.

b Densities were measured for the solutions in this study and calculated for the Hoehr et al. solutions based on the reported solution composition.

### 2.3 Reactor production of ^99^Mo

To evaluate the Tc/Mo separation, molybdenum-99 was produced in the 5 MW pool-type MTR research reactor at McMaster University and added to the cyclotron-irradiated PMA solutions. For the production of ^99^Mo, PMA hydrate (503 ± 1 mg) was placed in a sealed polyethylene tube (Adanac, Canada) and irradiated at a thermal neutron flux of 5‧10^12^ n/cm^2^s in an ex-core position for 60 min using the pneumatic ‘rabbit’ system. After irradiation, the sample was left to decay for 24 h before transferring it to the hot cell for the separation experiments. A stock solution of ^99^Mo-containing PMA was prepared by dissolving the neutron-activated PMA in distilled, deionised water to a concentration of 0.46 mol/L.

### 2.4 Mo/Tc separation evaluation

**Fig. 3:**
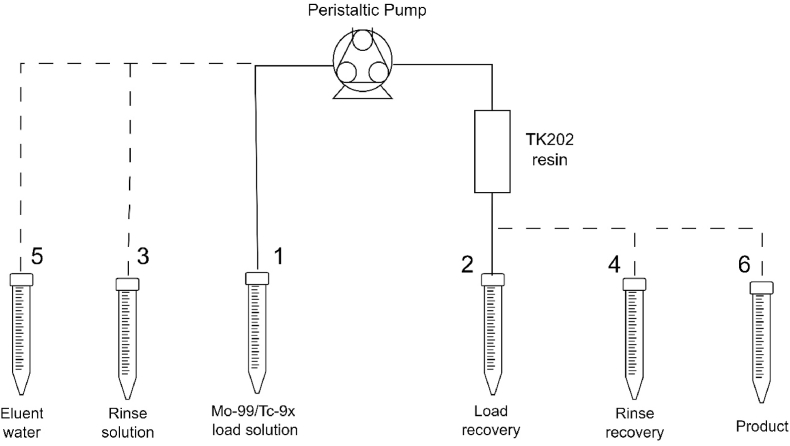
Schematic representation of Mo/Tc separation setup. The basic ^99^Mo/^9x^Tc load solution (1, details in Table 5) was pushed through the TK202 resin (25–352 mg) column at a flow rate of 0.5–1.2 mL/min using a peristaltic pump and collected in the load recovery tube (2). The resin was subsequently rinsed (3) with 3 mL of either sodium hydroxide (5 mol/L) or ammonium carbonate (2 mol/L) into the rinse recovery tube (4). Finally, the ^9x^Tc was eluted with 5 mL distilled, deionised water (5) into the product tube (6).

The aqueous biphasic TK202 resin (PEG-750 chain, 60–150 μm particle size, TrisKem International, France) was evaluated for the separation of technetium and molybdenum in the irradiated PMA solutions. The resin (25–352 mg/column) was dry-packed into 1 mL polypropylene SPE columns with frits (Sigma Aldrich, Canada). Prior to the separation experiments, the columns containing TK202 resin were conditioned with either sodium hydroxide (5 mol/L, 3 mL) or ammonium carbonate (1 mol/L, 3 mL) solution. The ^99^Mo-containing PMA stock solution (20–100 μL) was added to the cyclotron-produced ^9x^Tc PMA samples (400–2000 μL) before adding either an equal volume of sodium hydroxide (5–7 mol/L) or twice the volume of ammonium carbonate (2 mol/L), turning the solution basic (pH > 10). The basic ^99^MoO_4_^2−^/^9x^TcO_4_^-^ load solution was pushed through the TK202 resin column (flow rate 0.5–1.2 mL/min) using a peristaltic pump (Pulsafeeder Mec-o-matic, United States) according to the schematic representation in Fig. 3. The resin was then rinsed with either sodium hydroxide (5 mol/L, 3 mL) or ammonium carbonate (1 mol/L, 3 mL) solutions prior to the ^9x^TcO_4_^-^ being eluted with distilled, deionised water (5 mL) (see Fig. 3). The radioactive content in the pre-load, recovered load, rinse and product solutions, as well as the TK202 resin columns after separation, was analysed by gamma spectroscopy using the HPGe detector.

## 3 Results and discussion

### 3.1 ^94m^Tc production

All radioactive quantities reported are decay-corrected to the end of bombardment (EOB). In addition to the production of ^94m^Tc, the 12.9 MeV proton irradiation of the natural abundance molybdenum yielded several other technetium radionuclides (^94g^Tc: t_½_ = 293 min, ^95m^Tc: t_½_ = 61 d, ^95g^Tc: t_½_ = 20.0 h, ^96m^Tc: t_½_ = 51.5 min, ^96g^Tc: t_½_ = 4.28 d, ^99m^Tc: t_½_ = 6.01 h) in activity percentage ratios (Table 3) corresponding to those previous reported for the competing ^nat^Mo(p,x)^9x^Tc nuclear reactions (Červenák and Lebeda, 2016; Hoehr et al., 2012; Lamere et al., 2019; Tárkányi et al., 2012). Other than the Tc radionuclides, ^13^N (t_½_ = 9.97 min) was additionally produced by the ^16^O(p,α)^13^N reaction in the aqueous solutions. The use of enriched ^94^Mo in future evaluations will result in a more selective production of ^94m^Tc through the ^94^Mo(p,n)^94m^Tc reaction with the main co-produced impurity being ^94g^Tc from the ^94^Mo(p,n)^94g^Tc reaction (Bigott et al., 2006; Qaim, 2000). The results presented here mainly focus on the ^94m^Tc production and where appropriate are compared with data for the co-produced ^95g^Tc and ^96g^Tc (Table 4).

**Table 3:** The nuclear reaction and the average percentage activity at EOB (relative to ^94m^Tc defined as 100%) of the different Tc radioisotopes produced in the irradiations of natural-abundance PMA (n = 20) at 12.9 MeV. The isotope half-life, gamma identification lines and branching ratio are from National Nuclear Data Center (2024)

Nuclear reaction	Half-life (min)	Identification line (keV)	Branching ratio	Activity ratio (%)
^94^Mo(p,n)^94m^Tc	52.0	993.2	0.022	100
^94^Mo(p,n)^94g^Tc	293	702.7	0.996	5.1 ± 0.5
^95^Mo(p,n)^95m^Tc	87840	835.1	0.266	0.05 ± 0.01^a^
^95^Mo(p,n)^95g^Tc	1200	765.8	0.938	9.4 ± 1.1
^96^Mo(p,n)^96m^Tc	51.5	778.2^b^	0.019	447 ± 65^b^
^96^Mo(p,n)^96g^Tc	6163.2	812.5	0.820	2.7 ± 0.5
^100^Mo(p,2n)^99m^Tc	360.6	140.5	0.890	4.1 ± 0.5

a Average of seven irradiations.

b Activity of ^96m^Tc was calculated from area of the unique line of the ground state (812.5 keV): ^96m^Tc activity = area (778.2 keV) – area (812.5 keV) averaged over 13 irradiations.

**Table 4:** Experimental ^94m^Tc saturation yield (A_sat_) from the PMA evaluations at 12.9 MeV compared to the previously reported yields for AHM solutions A and C at 12.0 MeV published by Hoehr et al. (2012). The percentage of the calculated theoretical A_sat_ was based on cross section data (Červenák and Lebeda, 2016; Lebeda and Pruszyński, 2010; Tárkányi et al., 2012) from the EXFOR database (Zerkin and Pritychenko, 2018).

Solution	Irradiations performed	Proton range (mm)^a^	^94m^Tc experimental A_sat_ (MBq/μA)	Ratio of ^94m^Tc theoretical A_sat_ (%)	^95g^Tc experimental A_sat_ (MBq/μA)	^96g^Tc experimental A_sat_ (MBq/μA)
This work A	n = 16	1.77	20 ± 1	53.9 ± 3.4	38 ± 3	55 ± 7
This work B	n = 4	1.58	53 ± 6	52.8 ± 5.8	99 ± 11	143 ± 22
Hoehr et al. A	n = 2	1.54	16 ± 1	56.4 ± 3.5	–	–
Hoehr et al. C	n = 2	1.23	40 ± 6	56.6 ± 8.5	–	–

a Proton range was estimated by the SRIM program (Ziegler, 2013) based on the Mo solution compositions listed in Table 2 for the 12.9 MeV protons in this study and reported by Hoehr et al. (2012) for 12.0 MeV protons.

Table 4 summarises the saturation yields of the produced ^94m^Tc at EOB for the different PMA concentrations and volumes. Theoretical saturation yields were calculated using cross section data from the EXFOR database (Zerkin and Pritychenko, 2018) and the stopping power estimated by the SRIM program (Ziegler, 2013) based on the Mo solution compositions listed in Table 2. The measured saturation yields for ^94m^Tc were 20 ± 1 MBq/μA and 53 ± 6 MBq/μA for the PMA solution A and B, respectively, corresponding to 53.9 ± 3.4% and 52.8 ± 5.8% of the calculated theoretical yield. This correlates well with the ^94m^Tc yields reported by Hoehr et al. (2012) for their AHM solution A and C, which were 56.4 ± 3.5% and 56.6 ± 8.5%, respectively, of the calculated theoretical yield (Table 4). In this study, the measured saturation yields for ^96g^Tc and ^95g^Tc were 55 ± 7 MBq/μA and 38 ± 3 MBq/μA for PMA solution A and 143 ± 22 MBq/μA and 99 ± 11 MBq/μA for PMA solution B, respectively (Table 4), corresponding to percentages of the theoretical yields similar to that of ^94m^Tc with 56.5 ± 6.8% and 58.2 ± 4.2% in PMA solution A and 56.0 ± 8.5% and 57.9 ± 6.6% in PMA solution B for ^96g^Tc and ^95g^Tc, respectively. It is important to note that theoretical yields are based on EXFOR data from Mo solid targets, which assumes that the Mo concentration in the beam area is static. However, the setup is more complex for liquid targets where thermal and physical effects of the beam can induce convection and greatly change the composition and homogeneity of the target solution during irradiation, affecting the production yield (Do Carmo et al., 2020; Jahangiri et al., 2018; Krol et al., 2023; Pandey and DeGrado, 2021). Degradation of PMA by solution precipitation or formation of salt crystals in the target was not observed after irradiation (Fig. 1D). It is therefore expected that sub-processes such as heat-induced cavitation, water radiolysis-gas production and density change from phase-transition would be the main reason for the lower ^9x^Tc yields measured compared to the reported cross section data for the ^nat^Mo(p,x)^9x^Tc nuclear reactions (Table 4).

**Fig. 4:**
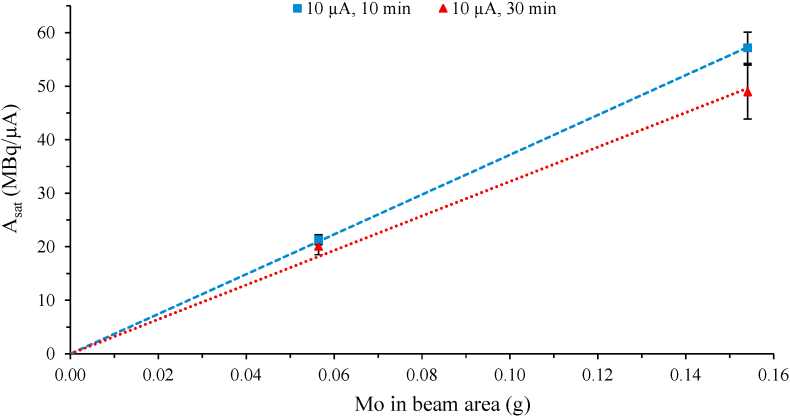
Mass of irradiated Mo in the beam area (15 mm diameter) versus ^94m^Tc saturation yield A_sat_, assuming liquid target is static.The line was a linear fit through 0,0 (x,y) for the 10 μA irradiations for 10 min (blue , R^2^ = 0.9994) with a slope of 372 MBq/(μA g). The longer irradiations (30 min) at 10 μA, especially at increased Mo mass in beam area, showed a less linear fit (red , R^2^ = 0.9900) when assuming a static liquid target, probably as the thermal and physical effects of the beam on the homogeneity of the target liquid would be increasing with irradiation intensity and time. (For interpretation of the references to colour in this figure legend, the reader is referred to the web version of this article.)

**Fig. 5:**
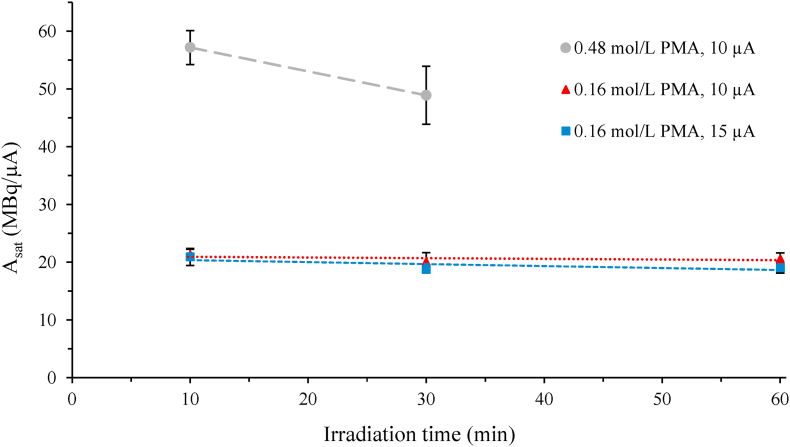
Saturation yield, A_sat_ (MBq/μA) as a function of irradiation time at 10 μA for (grey ) 0.48 mol/L PMA solution B and (red ) 0.16 mol/L PMA solution A, and at 15 μA for (blue ) 0.16 mol/L PMA solution A. While the PMA solution A had a constant A_sat_ of 20 ± 1 MBq/μA at the irradiation parameters evaluated here, the higher concentration PMA solution B (grey ) had decreasing A_sat_ with increasing irradiation time at 10 μA. Note that experiments at higher current and longer irradiation times were not carried out for solution B due to the operation limitations of the semi-automated delivery setup and increasing difficulty in target liquid recovery. (For interpretation of the references to colour in this figure legend, the reader is referred to the web version of this article.)

As seen in Fig. 4, the saturation yield was proportional to Mo mass in beam area for the 10-min irradiations at 10 μA. Increasing the irradiation time to 30 min resulted in a decrease in the saturation yield for the high concentration PMA solution B (Fig. 5). Note, irradiations >10 μA, >30 min were not carried out for solution B due to increasing difficulty in target liquid recovery associated with the operating limitations of the semi-automated target delivery setup, as described below. In contrast, a constant saturation yield was seen for the PMA solution A when increasing both the irradiation time and current up to 60 min at 15 μA. The PMA solution B experienced a more complete redox reaction during irradiation, as evidenced by a solution colour change to dark blue (Fig. 1C, discussed in more detail in next section), which could have affected the production yield. The maximum activity achieved for the PMA solution B was 161 ± 17 MBq ^94m^Tc for the irradiations at 10 μA for 30 min (n = 2), while a similar amount (157 ± 7 MBq ^94m^Tc) was produced for the PMA solution A at 15 μA for 60 min (n = 2). These results showed that the Mo concentration in PMA solution A was sufficient to produce ^94m^Tc amounts required for *in vivo* application. Using enriched ^94^Mo (>90%) instead of natural abundance Mo (^94^Mo 9.2%), yields up to 1.5 GBq ^94m^Tc can be expected.

### 3.2 Targetry

The system loading and delivery test with PMA solutions prior to irradiation resulted in an 88–93% recovery of the loaded volume in the product vial placed 5.6 m from the cyclotron (Fig. 2). The PMA product solution was delivered within 15 s at an argon pressure of 15–18 psi. The pre-irradiation test revealed that the majority of the volume loss was in the form of PMA droplets remaining in the loading line between the syringe and V2. In the irradiation experiments summarised in Table 1, comparable volume losses were observed for the PMA solution A with 83–91% volume recovery in the product vial. A slightly lower product volume recovery of 73–90% was observed for the high-concentration PMA solution B. Post-irradiation delivery was achieved with 18–22 psi argon pressure for the PMA solution A, while 22–25 psi argon was needed for the PMA solution B due to higher density and viscosity, thereby approaching the ≤30 psi pressure limit of the Burkert valves (V1, V2). The readout from the diode radioactivity detector placed at the product vial showed that the activity was being delivered as a bolus within 10–20 s. Only in the delivery of the product from the 30-min irradiations (at 10 μA) of the PMA solution B were droplets remaining in the last 50 cm of the delivery line, which then ended up in the vial of the first rinse (Table 1). In all other experiments ≥94% of the total activity was successfully transferred into the product vial.

The initial target volume loading evaluations showed that 2.3 mL of PMA solution was needed to cover the full beam area (15 mm width) in the 10 mm deep niobium body (Fig. 1A). For the irradiations, an adjusted loading volume of 2.6 mL PMA solution was chosen to account for the losses observed in the loading line and the potential losses due to evaporation during irradiation. Subsequent evaluations with 2.3 mL and 1.9 mL of PMA solution A did not result in lower production saturation yield, giving 22 MBq/μA and 20 MBq/μA, respectively, compared to the average 20 MBq/μA for all irradiations of PMA solution A (Table 4). This indicated that the collimated beam profile aligned on the target solution was more focused than the maximum 15 mm diameter beam area, defined by the width of the target beam line. Further reducing the PMA loading volume (<1.9 mL) in the Nb25 target body would likely decrease the production yield as the solution would start to not cover the beam profile (Fig. 1A). The target body used had a 10 mm deep cavity, but as seen in Table 4, the calculated 12.9 MeV proton range in the PMA solution was only 1.8 mm. A liquid target cavity depth of 5 mm might therefore suffice and would reduce the loading volume to around 1.2 mL PMA. Additional evaluation would, however, be required as these calculations are based on a static target material. The liquid target is, as previously discussed, more complex with liquid-vapour phase-change, which could affect density and cavitation formation and increase the proton range in the material. Optimisation of the target solution volume is essential to minimise the amount of costly enriched ^94^Mo material needed for each production and reduce the amount required to be separated in the product purification process.

Hoehr et al. (2012) reported formation of salt crystals in the target during AHM irradiation, which led to clogging of the valves. In contrast, no issues with PMA crystallising and blocking the target, lines or valves were experienced during the evaluations reported herein. The water rinses of the system after product delivery were very efficient due to the excellent water solubility of PMA. Any residues of PMA remaining in the target, valves and lines after the product delivery were captured in the first rinse vial with only traces (<0.05%) of the total activity in the second and third water rinse of the target (Table 1). The target was disassembled after approximately every third irradiation to assess the target niobium body and foil for any visible degradation. Throughout all experiments the niobium body and foils showed no signs of degradation and no traces of PMA precipitation or crystal formation (Fig. 1D). The same niobium foil was used in the reassembled target for all PMA solution A irradiations, while the foil was replaced with a new one for the PMA solution B experiments as a precaution.

In the experimental setup reported herein, the target was irradiated as an open system (as shown in Fig. 2) to evaluate the production yield without target pressure build up. For all irradiations, no target solution residues were observed in the waste vial before the gas trap. In the irradiations of PMA solution A at 15 μA for 60 min, droplets were observed in the first part of the line from the target to the gas trap. The initial delivery tests performed with non-irradiated PMA solution from the target to the hot cell in the laboratories above the cyclotron, via a 20 m line connected to V2 (Fig. 2), showed that >35 psi helium pressure was required to deliver the solution within 10 min. This was above the pressure limit of the solenoid valves (V1 and V2) used in this simple target loading and delivery setup. For future experiments, a fully automated system applying valves with a higher pressure tolerance would be required to evaluate the potential effects of a closed target system and pressure build up on the production yield. This automated system would also enable the transfer of the PMA solution from the target to the hot cell by applying a higher gas pressure for target emptying. Additionally, by placing the syringe loading driver closer to the target, the PMA volume lost in the loading line would be minimised, which is important when using the enriched ^94^Mo solution.

### 3.3 PMA oxidation states and Mo speciation

Fig. 1A–C presents the different colours of the PMA samples during the experiments. The yellow-coloured PMA complex (H_3_PMo(VI)_12_O_40_) (Fig. 1A, UV–vis absorption at 220, 315 and 420 nm) is composed of molybdenum(VI) oxide moieties arranged around a central phosphorus atom. PMA is generally very sensitive to reduction and easily converted to different derivatives of the green to dark blue mixed-valence phosphomolybdenum blue (H_4_PMo(VI)_8_Mo(V)_4_O_40_^3−^, PMB) (Nagul et al., 2015; Wu, 1920), which retains the PMA structure with delocalised electrons. The reduction of PMA is concentration-dependent and occurs by single electron steps in diluted concentrations (<0.1 mol/L) (Maksimovskaya, 2013), and in two-electron steps at higher concentrations. PMB complexes, having characteristic strong UV–vis absorption lines in the 700–900 nm range that increase in intensity as the colour changes from green to blue, can readily be re-oxidised by ambient oxygen if the reductant is not present in excess (Nagul et al., 2015). The irradiation of PMA solution A yielded a green-coloured product at EOB (Fig. 1B), which in all cases re-oxidised back to yellow within 2 h when stored under air in the collection vials. In contrast, the dark blue product of the irradiated PMA solution B remained blue (Fig. 1C, strong UV–vis: 725 nm) when stored for months under the same conditions. The chemistry occurring in a liquid target during irradiation is very complex due to the energies and temperatures involved, and several co-occurring processes that could initiate redox reactions, including electrochemical, photochemical and especially radiation induced reactions (Nagul et al., 2015; Wardman, 2023) likely contribute to the reduction of PMA. In particular, the formation of free radicals such as superoxide from water radiolysis in aqueous solutions could lead to the reduction of PMA. Additionally, the niobium metal is known to form thin oxide films (NbO, NbO_2_, Nb_2_O_5_) on its surface by two-electron donation steps when in the presence of water or other oxidising solutions (Sutherlin and Graham, 2005). Post-irradiation, the PMA product solutions were removed from these main sources (proton beam and niobium target) of reducing equivalents. However, in the case of PMA solution B, which remained blue for months (Fig. 1C, strong UV–vis: 725 nm), the more complete reduction of PMA to PMB suggest that an added oxidising agent is necessary to favour the re-oxidation.

Similarly, the solid PMA irradiated in the reactor for production of ^99^Mo turned greenish-blue (moderate UV–vis absorbance at 725 nm), likely due to radiation, photochemical and thermally-induced reduction. The neutron-activated reduced PMA stayed dark green upon dissolution in water, but its yellow colour returned within 60 s when added to the yellow cyclotron-irradiated PMA solution, possibly contributing to re-oxidising it. Additionally, the greenish-blue PMB solutions could be re-oxidised to yellow by addition of hydrogen peroxide. In any case, the reduction level of the irradiated PMA had no effect on the Mo/Tc separation efficiency, discussed in the next section. By the addition of base to the cyclotron- and reactor-irradiated PMA mixture (pH > 10, Table 5), the solution turned clear and colourless presumably as a result of decomposition of PMA into molybdate (Mo(VI)O_4_^2−^). Likewise, under basic conditions the technetium forms the pertechnetate necessary for the separation chemistry.

**Table 5:** Separation of ^99^Mo/^9x^Tc on TK202 resin. ^9x^Tc recovery from ^99^Mo/^9x^Tc solutions added either sodium hydroxide (NaOH) or ammonium carbonate ((NH_4_)_2_CO_3_).

Base added PMA mixture pre-separation^a^	Reactor ^99^Mo PMA (mL)	Cyclotron ^9x^Tc PMA (mL)	Total volume (mL)	^99^Mo/^nat^Mo ratio (kBq/mg)	TK202 resin (mg)	^nat^Mo/resin ratio (w/w)	^9x^Tc recovery of total (%)
NaOH Ratio 1:1 (v/v) (n = 7)	0.02	0.40 (n = 3)	0.84	0.88 0.69 0.69	25.3 51.2 102.6	3.28 1.62 0.81	51.9 80.0 96.8
	0.10	1.50 (n = 2)	3.20	0.68–0.72	197 ± 1	1.24 ± 0.08	99.2 ± 0.5
		2.00 (n = 2)	4.20	0.42–0.88	337 ± 23	1.61 ± 0.08	98.5 ± 0.3
(NH_4_)_2_CO_3_ Ratio 1:2 (v/v) (n = 3)	0.02	0.40 (n = 3)	1.26	0.88 0.69 0.69	25.5 51.1 102.2	3.25 1.62 0.81	20.8 59.3 94.8

a NaOH (5 mol/L) was added in equal volume and (NH_4_)_2_CO_3_ (2 mol/L) in double volume.

### 3.4 Tc/Mo separation

Aqueous biphasic extraction chromatographic (ABEC) resins are based on the separation technique developed for aqueous polyethyleneglycol (PEG) liquid/liquid extraction. In these systems, salts of water-structuring anions, such as PO_4_^3−^, CO_3_^2−^ and OH^−^ as well as molybdate, salt-out PEG and partition to the salt-rich phase, while the chaotropic anions like pertechnetate extract into the PEG-rich phase (Bond et al., 1999; Spear et al., 2000). The salting-out ability of the inorganic salts is related to Gibbs free energy of hydration of their anion and to a lesser extent the cation (Rogers et al., 1996). Higher concentrations of PEG or phase-forming salt (e.g. molybdate or hydroxide) will increase the separation into the two phases. The ABEC resins have covalently bound PEG chains acting as the solid separation support extracting pertechnetate from aqueous solutions while molybdate is not retained. Trace metals, such as Nb, Ni, Co and Zr, are additionally separated from the pertechnetate on the ABEC resins during the loading step together with the molybdate (Morley et al., 2012), which may impact the recycling process of the molybdenum target material. The pertechnetate can subsequently be stripped from the PEG chains of the ABEC resin by eluting with water, which decreases the concentration of water-structuring anions and thereby breaks down the aqueous biphasic system.

**Fig. 6:**
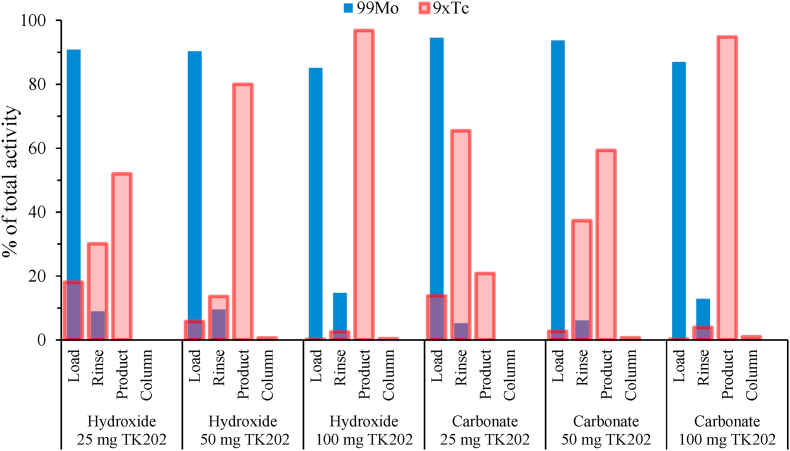
Separation of Mo and Tc from aliquots of irradiated PMA. Columns with 25, 50, and 100 mg TK202 resin were evaluated using either sodium hydroxide (5 mol/L) or ammonium carbonate (2 mol/L) mixed with the ^99^Mo/^9x^Tc PMA load solutions. The results are shown as the percentage distribution of ^99^Mo (blue) and ^9x^Tc (red) in the *load* solution recovery, *rinse* and *product* collection vials and remaining on the *column* with TK202 resin after the ^99^Mo/^9x^Tc separation. (For interpretation of the references to colour in this figure legend, the reader is referred to the web version of this article.)

Hoehr et al. (2012) reported that their concentrated AHM solutions prevented the retention of ^9x^Tc when using ABEC-2000 resin (PEG-2000 chain, 100–200 mesh, Eichrom Technologies, United States) with sodium hydroxide, while improved pertechnetate retention was obtained using ammonium carbonate (2 mol/L), although yielding a reduced recovery of 70%. For PMA, it was therefore decided to compare the effect of both the sodium hydroxide and ammonium carbonate anions on the TK202 resin (PEG-750 chain) separation of molybdenum and technetium using aliquots of the PMA solutions (Table 5, Fig. 6). Optimal retention of pertechnetate was reported by the supplier of the TK202 resin using 5–7 mol/L sodium hydroxide in the load and rinse steps (TrisKem International, 2021). An initial evaluation of sodium hydroxide concentration yielded no increased recovery of ^9x^Tc with 7 mol/L compared to 5 mol/L sodium hydroxide, therefore, the separation experiments listed in Table 5, were performed with 5 mol/L sodium hydroxide. To obtain a pH ≥ 10 for the complete conversion of molybdenum and technetium into their respective metalate species (MoO_4_^2−^ and TcO_4_^−^) an equal volume of sodium hydroxide (5 mol/L) was added, whereas twice the volume of ammonium carbonate (2 mol/L) was necessary. The ^9x^Tc product was eluted from the TK202 resin with 5 mL water.

Fig. 6 shows the distribution of ^99^Mo and ^9x^Tc in the load, rinse and elution steps when using 25, 50 and 100 mg TK202 resin, corresponding to a 3.3, 1.6 and 0.8 Mo/resin (w/w) ratio, respectively. Compared to the ammonium carbonate, a slightly better, although not optimal, ^9x^Tc retention (≤80%) was observed for the sodium hydroxide at Mo/resin (w/w) ratios ≥1.6. For the ammonium carbonate experiments, the majority of the ^9x^Tc loss was observed during the ammonium carbonate (1 mol/L) rinse step (Fig. 6). Good ^9x^Tc retention (>95%) was found for both the sodium hydroxide and ammonium carbonate load solutions at the 0.8 Mo/resin (w/w) ratio. In all separations, no ^99^Mo was detected in the ^9x^Tc product eluted with water (5 mL). As seen in Fig. 6, the majority of ^99^Mo (>85%) was separated during the loading step and the remaining activity removed during the rinse step. This is very important for future recycling of the target material when using enriched ^94^Mo. The retention of ^9x^Tc on the TK202 column after the product elution with water was less than 1%.

Due to the lower volume of sodium hydroxide required, and thereby lower resin loading volume, the scaled-up experiments were performed only with sodium hydroxide and not ammonium carbonate. If necessary, the separation using ammonium carbonate could also be optimised and applied for future work. Table 5 shows the results of the scaled-up experiments for PMA product volumes (1.5 mL and 2.0 mL). In both cases, the separation resulted in excellent ^9x^Tc recovery of >98% with no traces of ^99^Mo in the product. The high separation efficiency of PMA when using ABEC resin is suspected to be due to the presence of phosphate anion, in addition to molybdate, in the basic PMA solutions; phosphate has a large Gibbs free energy of hydration, especially compared to the AHM ammonium cation (Marcus, 1991). When scaling from the initial experiments with TK202 resin amounts of 100–300 mg (for 2.0 mL PMA), high back pressure was experienced during the loading step at a 1.2 mL/min flow rate. For the subsequent experiments using 200 mg resin (for 1.5 mL PMA) the flow rate was reduced to 0.5 mL/min (Table 5). The reduced flow rate had no effect on the separation efficiency, but increased the total processing time from 11 to 23 min. Fractions were collected to evaluate the water elution profile for the 200 mg TK202 resin separations, which showed that >98% of the activity was eluted in the first 3 mL and >95% in the first 2 mL at a flow rate of 0.5 mL/min. The eluted product had a pH of 10, necessitating further formulating prior to labelling. As previously reported, the pH adjustment can be achieved with a cation exchange resin, while an acidic alumina resin can concentrate and convert the pertechnetate into a saline solution (IAEA, 2017; Morley et al., 2012). In future evaluations, the separation process will be further optimised with a view to recycling of ^94^Mo, reducing column backpressure, further concentrating ^94m^Tc activity, optimising formulation prior to labelling, and minimising processing time when adapting the setup onto an automated system.

## 4 Conclusion

PMA as a target material for the production of ^94m^Tc offers significant advantages over state-of-the-art production methods and affords the potential to use liquid targets that are already installed at many medical facilities for clinical ^18^F production, enhancing the accessibility of the relatively short-lived ^94m^Tc for *in vivo* evaluation. Compared to the previous reported liquid target method for ^94m^Tc production (Hoehr et al., 2012), the better production yield, in-target solution stability during irradiation and ^94m^Tc separation recovery found for PMA makes it a very promising target material for routine clinical ^94m^Tc production. Of the concentrations evaluated in this study, the PMA solution A (0.16 mol/L, 0.181 g Mo/mL) gave the best results with a stable saturation yield of 20 ± 1 MBq/μA throughout the production parameters evaluated (10–60 min, 10–15 μA, 1.9–2.7 mL), yielding a maximum activity of 157 ± 7 MBq ^94m^Tc with irradiation at 15 μA for 60 min. Reduction of PMA was observed to varying degrees during irradiation, but this did not affect the post-irradiation separation of Mo and Tc, which yielded an excellent ^94m^Tc recovery (over 98%) using an ABEC resin. The production yield can likely be further optimised for the PMA solution A at higher beam current. Furthermore, by using enriched ^94^Mo PMA target solutions, yields up to 1.5 GBq ^94m^Tc should be achievable.

The target niobium body and cover foil showed good corrosion resistance throughout the PMA experiments. A minimum loading volume of 1.9 mL was necessary to cover the maximum beam area of the Nb25 target having a 10 mm deep cavity, however, the calculated 12.9 MeV proton range in the PMA solution (when assumed to be static) was less than 2 mm. Moving forward, a niobium cavity with a reduced depth, significantly lowering the PMA volume needed and thus the amount of enriched ^94^Mo required, will therefore be designed to evaluate its effect on the production yield. The target design is incorporated into a fully automated liquid target loading and delivery system, which can be tested as an open and closed system during PMA irradiation, to determine the parameters for the maximum achievable yield. Finally, the target solution will be delivered directly to an automated module for the ^94m^Tc purification, formulation and subsequent labelling of tracers to be evaluated in preclinical studies.

## CRediT authorship contribution statement

**Ross Harper:** Writing – original draft, Validation, Supervision, Resources, Project administration, Methodology, Investigation, Conceptualization. **Derek R. Morim:** Writing – review & editing, Validation, Methodology, Investigation. **Dhyey Mehta:** Writing – review & editing, Visualization, Investigation. **Veronika Rosecker:** Writing – review & editing, Methodology, Conceptualization. **Stephen J. Archibald:** Writing – review & editing, Funding acquisition. **Richard Southworth:** Writing – review & editing, Funding acquisition. **Philip J. Blower:** Writing – review & editing, Supervision, Funding acquisition. **Karin A. Stephenson:** Writing – review & editing, Supervision, Resources. **Karin M. Nielsen:** Writing – original draft, Visualization, Validation, Supervision, Project administration, Methodology, Investigation, Funding acquisition, Formal analysis, Conceptualization.

## Declaration of competing interest

The authors declare that they have no known competing financial interests or personal relationships that could have appeared to influence the work reported in this paper.

## Data Availability

Data will be made available on request.
